# Platelet membrane potential: unable to pull the plug on sepsis

**DOI:** 10.1186/cc13869

**Published:** 2014-05-12

**Authors:** Daniel A Schaer, James Penn, Sugeet Jagpal, Amay Parikh

**Affiliations:** 1 Internal Medicine Division, Department of Medicine, Rutgers Robert Wood Johnson Medical School, 1 Robert Wood Johnson Place, P.O. Box 19, New Brunswick, NJ 08903-0019, USA; 2 Pulmonary and Critical Care Division, Department of Medicine, Rutgers Robert Wood Johnson Medical School, 1 Robert Wood Johnson Place, P.O. Box 19, New Brunswick, NJ 08903-0019, USA; 3 Division of Nephrology, Department of Medicine, Rutgers Robert Wood Johnson Medical School, 1 Robert Wood Johnson Place, P.O. Box 19, New Brunswick, NJ 08903-0019, USA

## 

In a recent issue of *Critical Care*, we read with interest the work of Gründler and colleagues [1] regarding platelet mitochondrial depolarization reflecting disease severity in patients with sepsis. It has been established that, regardless of the source of sepsis, microcirculatory dysfunction with increased permeability and increased reactive oxygen species is similar among pathogens and leads to multi-organ dysfunction [2].

The microcirculatory response in patients with sepsis clearly influences clinical outcome, and biomarkers to reliably assess this response have been difficult to elucidate. Interestingly, the authors in this study were able to show a correlation between platelet membrane potential and Acute Physiology and Chronic Health Evaluation II and Sepsis-related Organ Failure Assessment scores and Simplified Acute Physiology Score II.

Several confounders such as neurodegenerative diseases and exposure to environmental agents such as cigarette smoke are known to affect platelet mitochondrial function [3]. It is difficult to control for patients with these confounders, and this could have biased the results of this study.

It is unclear what testing for platelet membrane potential adds to our ability to predict overall clinical outcome. Thrombocytopenia is already an established correlate to poor outcome in sepsis, and a platelet count, unlike the platelet membrane potential, is almost always readily available [4]. The authors did not control for thrombocytopenia, and it is unclear whether membrane potential would predict mortality, independent of platelet count.

We feel that this biomarker will not be useful in the clinical realm, but this work will add to our overall understanding of the microcirculatory physiologic response in sepsis.

## Competing interests

The authors declare that they have no competing interests.
